# Immune Cell Infiltrates and Neutrophil-to-Lymphocyte Ratio in Relation to Response to Chemotherapy and Prognosis in Laryngeal and Hypopharyngeal Squamous Cell Carcinomas

**DOI:** 10.3390/cancers13092079

**Published:** 2021-04-25

**Authors:** Mario Sánchez-Canteli, Luis Juesas, Esther Redin, Alfonso Calvo, Fernando López, Aurora Astudillo, Luis M. Montuenga, Juana M. García-Pedrero, Juan P. Rodrigo

**Affiliations:** 1 Department of Otolaryngology, Hospital Universitario Central de Asturias and Instituto de Investigación Sanitaria del Principado de Asturias (ISPA), 33011 Oviedo, Spain; uo216751@uniovi.es (M.S.-C.); luisjuesas94@gmail.com (L.J.); lopezafernando@uniovi.es (F.L.); 2 Instituto Universitario de Oncología del Principado de Asturias, University of Oviedo, 33003 Oviedo, Spain; 3 Ciber de Cáncer, CIBERONC, 28029 Madrid, Spain; eredin@alumni.unav.es (E.R.); acalvo@unav.es (A.C.); lmontuenga@unav.es (L.M.M.); 4 Program in Solid Tumors, Center for Applied Medical Research (CIMA), Department of Pathology, Anatomy and Physiology, University of Navarra, 31008 Pamplona, Spain; 5 Navarra’s Health Research Institute (IDISNA), 31008 Pamplona, Spain; 6 Tumor Biobank of Principado de Asturias, Hospital Universitario Central de Asturias and Instituto de Investigación Sanitaria del Principado de Asturias (ISPA), 33011 Oviedo, Spain; astudillo@uniovi.es

**Keywords:** larynx, hypopharynx, squamous cell carcinoma, chemotherapy, tumor infiltrating lymphocytes, PD-L1, neutrophil-to-lymphocyte ratio

## Abstract

**Simple Summary:**

The role of the immune response to cancer is of increasing importance, with a determining role in the response to the treatments and prognosis of patients. In this work, we studied whether the immune response (local and systemic) can influence the treatment response and prognosis of patients with laryngeal and hypopharyngeal carcinoma receiving induction chemotherapy (ICT). We observed that the relationship between neutrophils and lymphocytes (NLR) in peripheral blood and PD-L1 expression in the tumor is related to ICT response and patient prognosis. The identification of new biomarkers related to the immune response may allow a better selection of treatments and the identification of potential therapeutic targets.

**Abstract:**

Our goal was to assess the correlation of immune parameters with the response to induction chemotherapy (ICT) in head and neck squamous cell carcinoma (HNSCC) patients. Pretreatment biopsies from 64 patients with HNSCC that received ICT were assessed for PD-L1 protein expression and density of CD8+ and FOXP3+ tumor infiltrating lymphocytes (TIL). In addition, the neutrophil-to-lymphocyte ratio (NLR) was calculated from pretreatment whole blood counts. In total, 55% of cases exhibited PD-L1 combined proportion score (CPS) positivity (≥1% stained cells). PD-L1 CPS positivity correlated with a high density of both CD8+ (*p* = 0.01) and FOXP3+ (*p* < 0.001) TILs. There was no correlation between PD-L1 expression or TIL density and NLR values. In univariate analyses, the absence of PD-L1 CPS expression (*p* = 0.042) and a high NLR (*p* = 0.034) were significantly correlated with response to ICT. Neither CD8+ TIL (*p* = 0.99) nor FOXP3+ TIL densities (*p* = 0.71) were associated with response to ICT. In multivariate analysis, only a high NLR was associated with response to ICT (HR = 4.06, 95% CI = 1.06–15.5, *p* = 0.04). In addition, a high NLR was also independently associated with lower disease-specific (*p* = 0.03) and overall survival rates (*p* = 0.04), particularly in the subset of patients who received definitive surgical treatment. These results suggest that NLR could emerge as a predictive biomarker of response to ICT.

## 1. Introduction

Despite recent advances in treatment modalities, the prognosis of patients with advanced head and neck squamous cell carcinomas (HNSCC) is still poor, with 5-year survival rates barely reaching 50% [1]. An additional problem in cases with advanced tumors located in the larynx and/or hypopharynx is that the surgical treatment generally consists of a total laryngectomy. This causes significant psychosocial and functional consequences and the deterioration of patients’ quality of life, because of the critical role laryngeal function plays in voice production, swallowing, and the presence of a permanent tracheostoma. To avoid this, in selected cases, combined chemotherapy and radiotherapy offers the potential for organ preservation, without compromising patient survival [2]. Sometimes, induction chemotherapy (ICT) is administered before radiation. It is thought that tumor response to ICT may help for decision making on choosing the next treatment. If there is a good response, chemoradiation (CRT) with organ preservation appears as the best treatment choice. However, if there is little or no tumor response, surgery may be needed. Nevertheless, since neoadjuvant chemotherapy is not free of side effects and response varies among patients, it would be of great interest to identify biomarkers to accurately predict treatment response, which may help to improve patient stratification and to better select the most adequate candidates to undergo a laryngeal preservation protocol. Unfortunately, such biomarkers have not been identified yet [3].

HNSCC is an immunogenic tumor type, in which the escape from the immune system becomes particularly important. Malignant cells can evade the immune surveillance through several mechanisms, including the immunological checkpoints [4]. The programmed cell death ligand 1 (PD-L1) is commonly upregulated on the surface of tumor cells and binds to the programmed death 1 (PD-1) expressed on tumor-infiltrating lymphocytes (TIL), eventually causing a T-cell tolerance. This represents one of the multiple mechanisms of immune evasion [5]. The tumor expression of PD-L1 has also been related to a better response to immunotherapy [6]. However, its utility to predict response to classical chemotherapy is unclear, and there is only one previous study available in HNSCC [7].

The immune response to the tumor, mainly reflected by a high infiltration of CD8+ T lymphocytes (cytotoxic T lymphocytes), has been widely associated with a better prognosis in nearly all analyzed tumor types [8], including HNSCC [7,9,10,11]. Although most of these studies were performed in surgically treated patients, a high number of infiltrating CD8+ T cells has also been associated with a better response to definitive CRT [12]. These authors speculate that the killing of tumor cells by CRT might release tumor-associated antigens and chemokines that stimulate an immune response, plausibly enhanced in those patients harboring a high infiltration of immune cells within the tumor microenvironment (TME) before cancer treatment starts. This hence suggest that a pre-existing immunologic response might enhance the effects of CRT.

By contrast, it has also been reported that immune infiltrates may have the opposite effect, favoring tumor development and progression. This may occur when both the tumor and its surrounding TME activate mechanisms of chronic inflammation. Inflammatory cells are a source of growth factors, cytokines, reactive oxygen and nitrogen species, and enzymes that modify the extracellular matrix, which favor the proliferation and survival of tumor cells, angiogenesis, cell migration and metastasis [13]. Systemically, this inflammatory response is manifested by an increase in circulating neutrophils, monocytes, and platelets. In addition, this state of chronic inflammation reduces the number of T-lymphocytes available, capable of acting against tumor cells [14]. Thus, the neutrophil-to-lymphocyte ratio (NLR) obtained from the peripheral blood count is used as a marker of the relationship between the inflammatory response and the immune response to cancer. A high NLR has consistently been associated with a poor prognosis in a wide variety of solid tumors [15], including HNSCC [16]. Beyond this, some recent studies have pointed out that the NLR could also be related with the response to chemotherapy [17]; however, there is little evidence to date supporting this, with only three published studies available for HNSCC [18,19,20].

The overall objective of this study was to analyze the significance of NLR in peripheral blood and the immune infiltrate profiles (i.e., number of CD8+ and FOXP3+ TIL and PD-L1 expression) in pretreatment biopsies from patients included in a laryngeal preservation protocol, and to establish their potential relationship with the response to induction chemotherapy, as well as the impact on patients’ prognosis.

## 2. Materials and Methods

### 2.1. Patients

This study concerns 64 patients with pathologically confirmed, resectable, and previously untreated stage III or IV squamous cell carcinoma of the larynx or the hypopharynx treated at the Hospital Universitario Central de Asturias between 2007 and 2016.

Epidemiological data were recorded, including age, sex, smoking, and alcohol consumption. Tumors were staged following the 8th edition of the UICC TNM classification. Staging included a complete physical examination, a direct laryngoscopy, and contrast enhanced computed tomography (CT). None of the selected patients had distant metastases at the time of diagnosis.

All patients were candidates for a total laryngectomy and were included in an organ-preservation protocol [21] that consisted of chemoselection with one cycle of ICT followed by CRT in responders. Briefly, patients received a single cycle of ICT consisting of cisplatin 100 mg/m^2^ plus 5-Fluorouracil (5-FU) 1000 mg/m^2^/day for 5 days. All patients were examined by CT 3 weeks after ICT treatment to measure the percent reduction in primary tumor growth according to RECIST criteria [22]. To select the definitive treatment, tumor response was defined by a decrease of at least 50% in the largest tumor dimension. Responders underwent definitive CRT (cisplatin 75 mg/m^2^/day, three cycles in 3-week intervals plus standard radiotherapy, minimum 70 Gy) and non-responders underwent surgery followed by radiotherapy if indicated.

### 2.2. Tumor Samples

Pretreatment biopsy specimens from the patients were retrospectively collected. Sample use and experimental procedures were performed in accordance with the Declaration of Helsinki. Written informed consent was obtained from all patients. Formalin-fixed paraffin-embedded (FFPE) tumor biopsies and data from donors were provided by the Principado de Asturias BioBank (PT17/0015/0023), integrated in the Spanish National Biobanks Network, and histological diagnosis was confirmed by an experienced pathologist. Samples were processed following standard operating procedures with the appropriate approval of the Ethical and Scientific Committees of the Hospital Universitario Central de Asturias and the Regional CEIm from Principado de Asturias (date of approval: 14 May 2019; approval number: 141/19, for the project PI19/00560).

### 2.3. Hematological Parameters

Complete blood counts (CBC) with differential counting and several biochemical indices, such as hemoglobin (g/dL), were routinely recorded before starting ICT. NLR was calculated by dividing the absolute number of neutrophils by the number of lymphocytes obtained from the CBCs.

### 2.4. Immunohistochemical Study

The pretreatment FFPE tumor biopsies were cut into 3 µm sections and dried on Flex IHC microscope slides (Dako, Santa Clara, CA, USA). The sections were deparaffinized with standard xylene and hydrated through graded alcohols into water. Antigen retrieval was performed using Envision Flex Target Retrieval solution, high pH (Dako). Staining was performed at room temperature on an automatic staining workstation (Dako Autostainer Plus, Dako, Glostrup, Denmark) using the following primary antibodies: anti-PD-L1 antibody (clone E1L3N; Cell Signaling Technology, Danvers, MA, USA) at 1:200 dilution, anti-CD8 (clone SP16, Neomarkers, Freemont, CA, USA) at 1:400 dilution, or anti-FOXP3 (clone 236A/E7, Abcam, Cambridge, UK) at 1:400 dilution. Detection was performed with EnVision Flex+ Visualization System (Dako Autostainer, Dako, Glostrup, Denmark) for 30 min at room temperature, and immunostaining was developed using diaminobenzidine. Sections were washed in water, counterstained with hematoxylin and dehydrated before being mounted in DPX mounting medium. Positive controls were placenta samples for PD-L1 expression, and normal tonsil samples for CD8 and FOXP3 expression. Negative controls with an omission of the antiserum from the primary incubation were also included. In addition, samples of normal squamous epithelium obtained from non-oncologic surgery were also used as a negative control.

All slides were reviewed by two experienced pathologists. For tumor PD-L1 expression, only the membrane staining was evaluated, and expression was scored as: negative PD-L1 expression (<1% stained cells), low PD-L1 expression (≥1–<10%), intermediate PD-L1 expression (≥10–<50%), or high PD-L1 expression (≥50%). Tumor proportion score (TPS) positivity was defined as ≥1% stained tumor cells based on current recommendations [6]. Combined proportion score (CPS), defined as the number of PD-L1-positive cells (tumor, lymphocytes, and macrophages) in relation to total tumor cells, was also considered positive if ≥1%.

Quantification of TIL staining (both CD8+ and FOXP3+) was performed automatically using ImageJ software, considering lymphocytes within the tumor but not the peritumoral area. Five high-power fields (200×) were counted per sample in the tumor area, and the total number of cells positive for each marker was expressed as density per mm^2^ and averaged. For statistical purposes, the mean values were used as cut-off points to define high and low density for both CD8+ and FOXP3+ TILs. Scoring was conducted blinded to clinical outcomes.

### 2.5. Statistical Analysis

Chi-squared and Fisher’s exact tests were used for comparisons between categorical variables. Receiver Operating Characteristics (ROC) curve analysis was used to determine the optimal cut-off point for NLR using the ICT response and the patient’s death due to the tumor as variables. Based on the area under the ROC curve, expressed with 95% confidence intervals (CIs) and *p* values, the Youden Index cutoff levels were determined. For time-to-event analysis, Kaplan–Meier curves were plotted. Cox proportional hazards models were utilized for univariate and multivariate analyses. The hazard ratios (HR) with 95% confidence intervals (CI) and *p* values were reported. All tests were two-sided. *p* values of ≤0.05 were considered statistically significant.

## 3. Results

### 3.1. Patient Characteristics

The characteristics of the patients are shown in Table 1. After one cycle of ICT, one patient showed progressive disease, five cases stable disease, 58 cases a partial response, and none showed a complete response. From the 58 patients with a partial response, 26 patients (41%) who showed a response to ICT greater than 50% in the larger dimension of the primary tumor were considered responders and followed treatment with concomitant CRT. The remaining patients received a total laryngectomy plus neck dissection (22 bilateral and 16 unilateral), and 17 cases underwent postoperative RT.

Response to ICT was not significantly associated with age (*p* = 0.32), gender (*p* = 1), tumor location (*p* = 0.45), stage of disease (*p* = 0.79), or histological grade (*p* = 0.89).

The mean follow-up of patients was 60 months (median 51 months, range 5–156 months). During the follow up, 22 patients (34%) presented tumor recurrence: four local, three regional, four local and regional, five distant metastases, and six loco-regional and distant metastases. In addition, 11 patients (17%) developed a second primary tumor. Most cases (8/11) presented a second tumor in the lung, and the other three were developed in the oral cavity, oropharynx, and a lymphoma.

The 5-year overall survival (OS) was 60%, and the 5-year disease-specific survival (DSS) was 70%. There were no significant differences in DSS (*p* = 0.96) or OS (*p* = 0.16) depending on the response to ICT, and, therefore, the type of definitive treatment either surgery or CRT (Supplementary Figure S1).

### 3.2. Hematological Parameters and Response to ICT

The results of the analyzed hematological parameters for the HNSCC patients before ICT treatment are shown in Supplementary Table S1. No significant differences were found in the levels of hematological parameters in relation to the response to ICT (ANOVA test, Table 2). Nevertheless, a higher number of neutrophils was observed in those patients who responded to ICT and a lower number of lymphocytes. Consequently, the mean NLR value was higher in responders compared to non-responders, the differences reaching borderline statistical significance (*p* = 0.058). The ROC curves showed that the optimal cut-off level for NLR was 4.5 (Supplementary Figure S2A). Using this cut-off point, we found that cases with high NLR presented a significantly better response to ICT (10/15, 67%) than those with low NLR (16/49, 33%) (Fisher’s test = 5.508; *p* = 0.034).

### 3.3. PD-L1 Expression and Response to ICT

**Figure 1:**
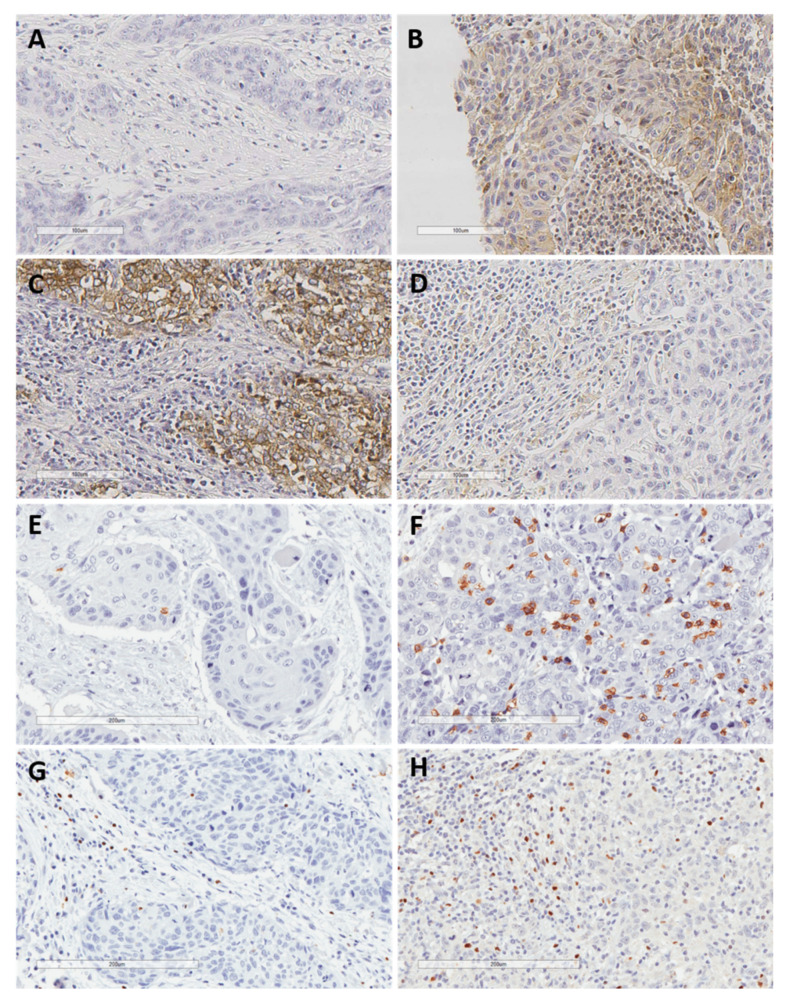
Representative examples of negative PD-L1 expression in both tumor and the surrounding immune cells (**A**), positive PD-L1 expression in both tumor and stromal immune cells (**B**), positive PD-L1 expression in tumor cells but not in surrounding immune cells (**C**), positive PD-L1 expression in immune cells but not in tumor cells (**D**), low and high density of CD8+ TIL (**E**,**F**), and low and high density of FOXP3+ TIL (**G**,**H**). Original magnification ×200.

In total, 44 cases (69%) exhibited negative PD-L1 TPS expression, 13 (20%) low expression, five (8%) intermediate expression, and only two cases (3%) high PD-L1 TPS expression. Regarding PD-L1 CPS, 29 cases (45%) showed negative expression, 26 (41%) low expression, six (9%) intermediate expression, and three (5%) high expression. Figure 1A–D display representative examples of PD-L1 expression in HNSCC biopsies prior to ICT.

PD-L1 TPS was not associated with response to ICT: 19/44 (43%) patients with negative PD-L1 TPS showed response to ICT versus 7/20 (35%) patients with positive PD-L1 TPS (*p* = 0.59). However, PD-L1 CPS positivity showed a significant association with a worse response to ICT: 16/29 (55%) patients harboring negative PD-L1 CPS responded to ICT treatment versus 10/35 (28.5%) cases with positive PD-L1 CPS expression (*p* = 0.042).

### 3.4. TIL Density and Response to ICT

TIL evaluation could be performed in all the 64 tumor biopsies from HNSCC patients prior to ICT (Table 3). Twenty cases (31%) were classified as high CD8+ TIL density and 24 cases (37.5%) as high FOXP3+ TIL density, dichotomized according to the mean TIL density. Representative examples of high and low density of TILs are shown in Figure 1E–H. There was a strong positive correlation between the density of both CD8+ and FOXP3+ TILs (Spearman’s Rho coefficient = 0.45, *p* < 0.001).

There were no differences in the mean density of TILs between the patient subgroups of responders and non-responders to ICT (Table 3). However, the mean ratio between CD8+ TIL and FOXP3+ TIL density was significantly higher in the subgroup of responders to ICT (*p* = 0.05). 

### 3.5. Relationship among NLR, PD-L1 Expression, and TIL Density

CD8+ and FOXP3+ TIL densities were both found to significantly correlate with positive PD-L1 CPS expression (Spearman’s Rho coefficient = 0.32, *p* = 0.01, and Spearman’s Rho coefficient = 0.239, *p* < 0.001, respectively).

Subsequently, PD-L1 CPS and CD8+ TIL densities were combined to categorize the immune response to the tumor into four subtypes, as described by Teng et al. [23]: positive PD-L1 CPS /high CD8+ TIL density (type I, adaptive immune resistance) was observed in 15/64 cases (23%); negative PD-L1 CPS /low CD8+ TIL (type II, immunological ignorance) in 24/64 cases (37%); positive PD-L1 CPS /low CD8+ TIL (type III, intrinsic induction) in 20/64 cases (31%); and negative PD-L1 CPS /high CD8+ TIL (type IV, immune tolerance) in 5/64 cases (8%). The type of immune response was next correlated with the response to ICT. We found that patients with type IV exhibited the highest response rate (80%), whereas only 20% of patients with type III responded to ICT, and types I and II showed intermediate responses, with nearly significant differences (*p* = 0.055; Table 4). Nevertheless, these results should be interpreted with caution, taking into consideration the limited number of patients in some groups.

The mean NLR was similar in positive PD-L1 TPS versus negative PD-L1 TPS cases (ANOVA test, *p* = 0.764). When assessed by PD-L1 CPS, patients with PD-L1 CPS positivity had a lower although not significant mean NLR (ANOVA test, *p* = 0.19). As expected, according to the data from our individual analysis, patients with high NLR and negative PD-L1 CPS significantly showed the best response to ICT, whereas those with low NLR and positive PD-L1 were the worst responders (Chi squared test, *p* = 0.035; Table 4).

Finally, there were no significant correlations between the NLR and the density of CD8+ TIL (Spearman’s Rho coefficient = −0.018, *p* = 0.89) or FOXP3+ TIL (Spearman’s Rho coefficient = −0.155, *p* = 0.25). Similarly, no association was observed between the CD8+/FOXP3+ ratio and the NLR (Spearman’s Rho coefficient = 0.075, *p* = 0.58).

Multivariate analysis (logistic regression) was performed to assess the independent predictive value of the immunological parameters analyzed in relation to the response to ICT (Table 5). The only parameter independently associated with the response to ICT was a high NLR value (HR = 4.06, 95% CI = 1.06–15.5, *p* = 0.04).

### 3.6. Relationship among NLR, PD-L1 Expression, TIL Density, and Patient Survival

**Figure 2:**
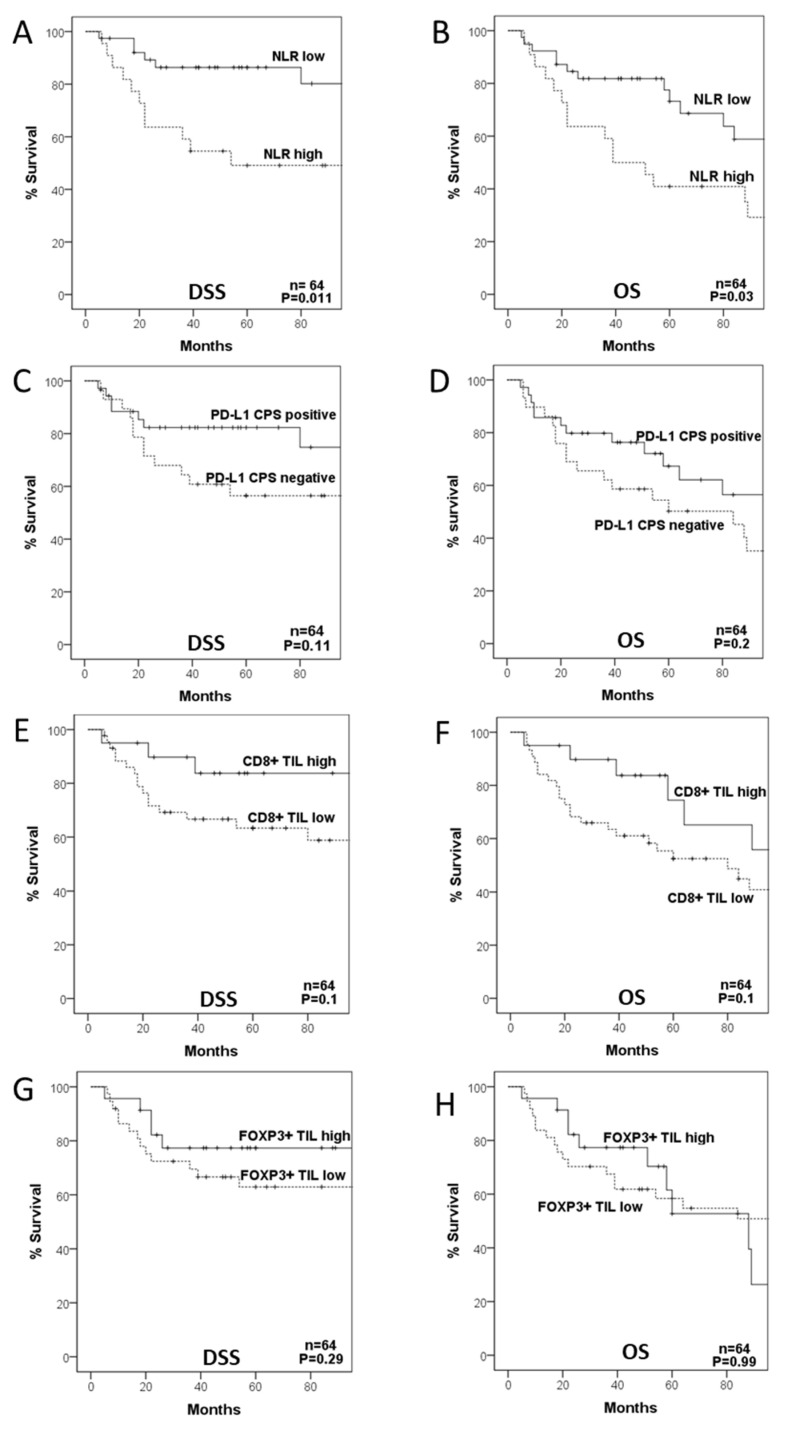
Disease-specific (DSS) and overall survival (OS) curves categorized according to neutrophil-to-lymphocyte ratio (NLR (**A**,**B**)), PD-L1 composite proportion score (CPS (**C**,**D**)), CD8+ TIL density (**E**,**F**), and FOXP3+ TIL density (**G**,**H**).

We next assessed possible relationships between the different parameters analyzed for the immune TME and patient survival. Analysis of the ROC curve revealed that the optimal cut-off point for the NLR was 3.86, which was established and used as a cut-off for subsequent analyses (Supplementary Figure S2B). Patients with high NLR exhibited a significantly worse OS and DSS than those with low NLR (HR = 2.26, 95% CI = 1.1–4.76, *p* = 0.03 and HR = 3.62, 95% CI = 1.34–9.18, *p* = 0.011, respectively; Figure 2A,B). According to the definitive treatment, it was observed that although the prognostic value of the NLR remained in patients treated with CRT, it was only significant in those surgically treated (HR = 2.26, 95% CI = 0.6–9.6, *p* = 0.25 and HR = 5.1, 95% CI = 1.2–21.3, *p* = 0.027, respectively; Supplementary Figure S3A,B).

Regarding PD-L1 CPS expression, patients harboring PD-L1 CPS positivity showed a better DSS and OS than cases with negative PD-L1 CPS expression, although the differences did not reach statistical significance (HR = 0.47, 95% CI = 0.18–1.19, *p* = 0.11, and HR = 0.62, 95% CI = 0.3–1.3, *p* = 0.2; Figure 2C,D). The relationship of PD-L1 CPS expression with a better prognosis was observed in patients treated with CRT but not with surgery (HR = 0.2, 95% CI = 0.02–1.6, *p* = 0.13 and HR = 0.67, 95% CI = 0.2–2.4, *p* = 0.54, respectively; Supplementary Figure S3C,D).

When evaluating the impact of CD8+ and FOXP3+TILs on patient survival, we found that patients with high CD8+TIL density had a better DSS and OS than those with low CD8+TIL, although the differences were only close to statistical significance (HR = 0.36, 95% CI = 0.1–1.2, *p* = 0.1 and HR = 0.48, 95% CI = 0.2–1.2, *p* = 0.1, respectively; Figure 2E,F), and with a similar trend in cases treated with either surgery or CRT (HR = 0.36, 95% CI = 0.07–1.7, *p* = 0.2 and HR = 0.29, 95% CI = 0.03–2.3, *p* = 0.24, respectively; Supplementary Figure S3E,F). FOXP3+ TIL density was not associated with DSS (HR = 0.57, 95% CI = 0.2–1.61, *p* = 0.29) or OS (HR = 0.99, 95% CI = 0.45–2.2, *p* = 0.99; Figure 2G,H). 

The immune TME subtype also significantly influenced patient survival, with type I having the best DSS and OS, and type II the worst survival rates (type I versus type II HR = 0.14, 95% CI = 0.02–1.1, *p* = 0.06 and HR = 0.31, 95% CI = 0.09–1.08, *p* = 0.06, respectively; Supplementary Figure S4). 

Multivariate Cox regression analysis, including tumor localization, disease stage, PD-L1 CPS, NLR, CD8+ TIL, and FOXP3+ TIL, further showed that the parameters independently associated with a worse DSS were hypopharyngeal localization (HR = 4.22, 95% CI = 1.32–13.48, *p* = 0.015) and a high NLR (HR = 3.16, 95% CI = 1.07–9.32, *p* = 0.03). Accordingly, the same parameters were also found to independently associate with a worse OS: hypopharyngeal localization (HR = 2.63, 95% CI = 1.17–5.89, *p* = 0.019) and high NLR (HR = 2.23, 95% CI = 1.01–4.92, *p* = 0.04).

Given the influence of tumor location on patient prognosis, we further analyzed the NLR values, PD-L1 CPS expression, and CD8+ TIL density according to tumor location. Although the NLR mean values were higher and the mean CD8+ TIL density and PD-L1 CPS positivity were lower in hypopharyngeal tumors, the differences were not significant (*p* = 0.4, *p* = 0.13, and *p* = 0.13, respectively; Table 6).

## 4. Discussion

Currently, there are no validated biomarkers to predict response to treatment with ICT in HNSCC, although some previous studies have explored this possibility [18,24]. In this work, we analyzed the predictive and prognostic capacity of several parameters related to the immune response to the tumor (i.e., NLR, PD-L1, CD8+, and FOXP3+ TILs) using a selected cohort of advanced HNSCC patients included in an organ-preservation protocol and treated with ICT. Our results revealed that the response to ICT was more favorable in patients with a high NLR (setting the cut-off point at 4.5) and in those with negative PD-L1 expression. Moreover, the combination of both parameters increased the predictive capacity: patients with high NLR and negative PD-L1 showed a higher response to ICT (78%) as opposed to patients with low NLR and positive PD-L1 expression (only 25% responded). By contrast, the density of lymphocyte infiltration was not related to the response to ICT; however, the CD8+/FOXP3+ TIL ratio was significantly higher in responders to ICT. Interestingly, although patients with a low NLR and those with positive PD-L1 had a lower response to ICT, these two subgroups were significantly associated with improved patient survival. Nevertheless, results from the multivariate analysis further showed that only the NLR value was independently associated with response to ICT and disease prognosis.

The NLR has been found to influence prognosis in multiple tumor types [15]. A high NLR value has been almost systematically associated with a worse prognosis, including HNSCC [16,25]. In accordance with this, we also found that a high NLR was independently associated with a shortened survival. In this study, the optimal cut-off point for prognosis was established at 3.8, which is in the range of those reported in previous studies, which varied between 3 and 5 [25]. An elevated NLR indicates systemic inflammation. The inflammatory response associated with cancer plays a key role in clinical outcomes [14,15,16,25]. Cancer cells recruit and activate leukocytes, and although the precise role of neutrophils within the TME remains controversial, they appear to facilitate tumor progression. Tumor-associated neutrophils (TAN) appear to contribute to tumor growth, angiogenesis, and immune tolerance [26]. The interaction between tumor cells and the surrounding immune TME allows immune edition, by which tumor cells act on immune tolerance through TAN leading to tumor growth [26]. In addition, tumor cells secrete neutrophil-attracting cytokines that contribute to the destruction of basement membranes, thereby facilitating invasion and lymphatic metastases [13]. Therefore, an inflammatory response to the tumor with the predominance of neutrophils (reflected as an elevated NLR) would be harmful, while an immune response with the predominance of lymphocytes and a low NLR should be more beneficial to the patient.

Previous data on the predictive value of NLR to the response to ICT are much scarcer. A recent meta-analysis [17] involving bladder, breast, rectum, and gastroesophageal tumors showed that a low NLR is associated with a better response to ICT and a better prognosis. However, unlike the studies on the prognostic value of the NLR, heterogeneity was greater in this meta-analysis, since some studies found a better response to ICT in patients with elevated NLR, such as a recent large study in HER-2 negative breast cancer patients (n = 1097) that also showed that a high NLR was an independent predictor of complete pathologic response after neoadjuvant chemotherapy [27]. Only three previous studies assessed the relationship between the NLR value and the response to ICT in HNSCC [18,19,20]. Two of them [18,20] did not find a significant correlation, and a third study [19] that included hypopharyngeal tumors and, as in our study, a high NLR was significantly associated with a better response rate to ICT. In all these studies and ours, an elevated NLR was concordantly associated with a worse prognosis, regardless of the response to ICT. These contradictory results may be explained and perhaps reflect the fact that the response to ICT (and thus the type of definitive treatment performed) is an independent characteristic not associated with disease prognosis. Accordingly, parameters that are associated with response to ICT could behave differently in relation to patient outcome. However, given the limited size of our series of patients and the highly heterogeneous results reported in the literature, we cannot exclude the possibility that the association observed between a high NLR and a better response to ICT in our HNSCC cohort may be due to chance. It is, however, noteworthy that analogous results to ours have also been reported in a large recent study on breast cancer patients [27]. Thus, these authors also found that an elevated NLR was associated with a better response to ICT and, at the same time, with poor survival outcomes, in good agreement with our data. 

The prognostic value of PD-L1 expression in HNSCC has been extensively studied, although with variable results [28], as occurred in many other solid tumors [29]. The discrepancies in results could be attributed to the use of different antibodies, various cut-off values of PD-L1 positivity, varying scoring criteria (including or not the immune cells), and also the intratumoral heterogeneity of PD-L1 expression [30]. In this HNSCC cohort, we observed that positive PD-L1 expression was significantly associated with higher DSS rates, especially when expression was measured in both tumor and immune cells (CPS) and in cases definitively treated with CRT. Nonetheless, no significant differences were reached, probably due to the relatively small sample size. Our findings are consistent with several recent studies on HNSCC that consistently showed the association of PD-L1 expression with increased survival in both tumors treated with surgery and CRT [10,11,31]. Moreover, in all these studies and ours, PD-L1 expression was strongly correlated with high cytotoxic CD8+ TIL density in the TME. Therefore, PD-L1 expression could represent a surrogate marker of the endogenous anti-tumor immune response, thus explaining its association with a good prognosis. Accordingly, a high density of CD8+ TIL was also associated with better survival rates in our study, although the differences did not reach statistical significance, again probably related to the sample size. High infiltration by CD8+ TIL has been found to be consistently associated with favorable outcomes in HNSCC, as demonstrated in a recent meta-analysis [9]. As PD-L1 positivity and a high density of FOXP3+ TIL were correlated with a high density of CD8+ TIL infiltration, this could jointly explain their association with better survival.

Interestingly, the survival benefit of patients with high CD8+ TIL density was commonly observed in patients either receiving surgery or CRT as definitive treatment. Most studies showing the better prognosis associated with a high TIL infiltration in HNSCC are performed in resected specimens, but in line with our findings, a previous study also found that a high number of infiltrating CD8+ T cells predicts the response to definitive CRT [12]. As mentioned, these authors suggest that a pre-existing immunologic response might enhance the effects of CRT.

Based on combined PD-L1 expression and CD8+ TIL density, a classification of tumors into four groups has been proposed [23]. According to this classification, type II tumors (low CD8+ TIL /negative PD-L1; immunological ignorance) were predominant (37%) in our study, whereas only 23% of patients were classified as type I tumors (high CD8+ TIL /positive PD-L1; adaptive immune resistance). These data are comparable to those reported by other HNSCC studies [10,11,29], which showed 14–20% type I tumors. Our findings are also in good agreement with these studies, commonly describing a strong positive correlation between PD-L1 expression and CD8+ TIL, and as a consequence, patients harboring positive PD-L1, high CD8+ TIL, and type I tumors concordantly showed a better prognosis [10,11,31].

By contrast, to date, few studies have investigated the predictive value of PD-L1 expression and TIL infiltration as markers of the response to ICT in HNSCC. Regarding PD-L1 expression, only a single study is yet available addressing this question [7], specifically, in hypopharyngeal tumors. This study reported no relationship between PD-L1 TPS expression and the response to ICT and no impact on patient prognosis. Analogously, we did not find a significant correlation between PD-L1 TPS expression and response to ICT. However, it is noteworthy that we found that PD-L1 CPS expression was inversely correlated with the response to ICT. This underscores the importance of considering both tumor and stromal cells for the evaluation of PD-L1 expression, as recommended in the recent guideline on immunotherapy in HNSCC [6]. However, we must keep in mind that the results are based on the analysis of pretreatment biopsies, and given the intratumoral heterogeneity of PD-L1 expression, this may not reflect the expression in the whole tumor [30]. In addition, as in the case of the NLR, the contradictory results observed between the predictive value of PD-L1 in relation to the response to ICT and its impact on prognosis and disease outcome, and the paucity of previous studies on the subject mean that the results should be interpreted with caution and will require confirmation in other series of patients. The same study [7] is also the only one to date to show a link between high CD8+ TIL density and improved response to ICT. Studies in other tumors (e.g., breast and rectum) have provided similar results, with tumors highly infiltrated by TIL having better response to ICT [32,33]. Even though we did not observe a significant association, patients with higher CD8+/FOXP3+ ratios (reflecting a predominance of CD8+ TIL) showed better responses to ICT. A similar study in non-small cell lung cancer also showed a better response rate to platinum-based ICT in patients with higher CD8+/FOXP3+ ratios (both in adenocarcinomas and squamous cell carcinomas), although CD8+ TIL infiltration was only associated with the response to ICT in adenocarcinomas [34]. These differences between studies could be influenced by the different tumor types and chemotherapy regimens or protocols used to evaluate TIL density, suggesting that the predictive value of TIL infiltration requires further validation.

Few studies have addressed the relationship among PD-L1 expression, TIL infiltration, and markers of systemic inflammation, such as the NLR. No relationship between the expression of NLR and PD-L1 has been reported in lung cancer [35,36], while a higher NLR has been related with PD-L1 positivity in bladder cancer [37]. In marked contrast, positive PD-L1 has been associated with a low NLR in esophageal cancer [38]. There is only one previous study on oral and pharyngeal cancer [39], which showed high NLR levels associated with PD-L1 positivity, in contrast with our herein findings. It would be expected that an immune response to the tumor, and, therefore, a low NLR, would be associated with a greater infiltration of T lymphocytes, which could explain their association with a better prognosis, as shown by some authors [40]. Even though low NLR and a high CD8+ TIL density were both associated with a better prognosis, we did not find a significant relationship between these two parameters, therefore indicating that the NLR could not be used as a surrogate marker of TIL infiltration in HNSCC, and more importantly, the NLR was found to be the strongest predictor of patient prognosis among the different parameters of immune TME jointly evaluated in our study.

## 5. Conclusions

In conclusion, both negative PD-L1 CPS expression and elevated NLR values were significantly associated with an improved response to ICT, with the best responders being those who combined high NLR and negative PD-L1. TIL infiltration (by either CD8+ or FOXP3+ T cells) was not predictive of a response to ICT. Although patients harboring positive PD-L1 and high CD8+ TIL infiltration showed improved survival, only the NLR was found as an independent predictor of HNSCC prognosis, with patients with low NLR having longer survival rates.

## Figures and Tables

**Table 1:** Clinicopathological characteristics of the included patients.

Characteristic	Number of Cases (%)
Age in years (mean; range)	59 (36–70)
Gender	
Male	58 (91%)
Female	6 (9%)
Tobacco consumption	
No	1 (2%)
Yes	62 (98%)
Not available	2
Alcohol consumption	
No	16 (26%)
Yes	46 (74%)
Not available	2
Localization	
Larynx	36 (56%)
Hypopharynx	28 (44%)
T classification	
T2	6 (9%)
T3	53 (83%)
T4	5 (8%)
N classification	
N0	28 (44%)
N1	11 (17%)
N2	22 (34%)
N3	3 (5%)
Stage	
III	37 (58%)
IV	27 (42%)
Histological grade	
G1	16 (27%)
G2	26 (44%)
G3	17 (29%)
Undetermined	5

**Table 2:** Mean values (±standard deviation) of the hematological parameters in responders and non-responders to induction chemotherapy (ICT).

Parameter	Responders	Non-Responders	*p* *
Hemoglobin (gr/dL)	14.56 ± 1.43	14.1 ± 1.78	0.28
Leucocytes (cells/mm^3^)	9065 ± 1993	8682 ± 1795	0.43
Neutrophils (cells/mm^3^)	6141 ± 2291	5628 ± 1645	0.31
Lymphocytes (cells/mm^3^)	1978 ± 1016	2155 ± 771	0.44
NLR	4.53 ± 4.1	3.1 ± 1.6	0.058

NLR: neutrophil-to-lymphocyte ratio. * ANOVA test.

**Table 3:** Tumor-infiltrating lymphocytes (TIL) count per mm^2^ in tumor samples and its relationship with response to induction chemotherapy.

TIL	Total Cases Mean/Median (Range)	Responders Mean	Non-Responders Mean	*p* *
CD8^+^ TIL (*n* = 64)	392/307 (4–1516)	392	392	0.99
FOXP3^+^ TIL (*n* = 64)	232/172 (4–796)	242	224	0.71
CD8^+^/FOXP3^+^ ratio (*n* = 64)	3.4/2.1 (0.1–24.8)	4.9	2	0.05

TIL: Tumor infiltrating lymphocytes. * ANOVA test.

**Table 4:** The type of immune response to the tumor and the combination of PD-L1 expression and neutrophil-to-lymphocyte ratio (NLR) with the response to induction chemotherapy.

Characteristic	Responders (%)	Non-Responders (%)	*p* *
Type of immune tumor microenvironment			
Type I	6 (40)	9 (60)	0.055
Type II	12 (50)	12 (50)	
Type III	4 (20)	16 (80)	
Type IV	4 (80)	1 (20)	
Combined NLR/PD-L1 CPS			
High NLR/positive PD-L1	3 (50)	3 (50)	0.035
High NLR/ negative PD-L1	7 (78)	2 (22)	
Low NLR/ positive PD-L1	7 (25)	21 (75)	
Low NLR/ negative PD-L1	9 (50)	9 (50)	

* Chi-squared test.

**Table 5:** Multivariate analysis in relation to the response to induction chemotherapy.

Parameter	Hazard Ratio	95% Confidence Interval	*p*
NLR			
<4.5	1		0.04
>4.5	4.06	1.06–15.5	
PD-L1 CPS			
Negative	1		0.1
Positive	0.397	0.13–1.19	
CD8+ TIL density			
Below mean	1		0.21
Above mean	2.38	0.62–9.13	
FOXP3+ TIL density			
Below mean	1		0.12
Above mean	2.71	0.76–9.74	

**Table 6:** NLR values, CD8+ TIL density, and PD-L1 CPS expression according to tumor localization.

Variable	Larynx	Hypopharynx	*p*
NLR mean	3.42	4.08	0.4 *
CD8+ TIL density mean (cells/mm^2^)	450	317	0.13 *
PD-L1 CPS positivity (%)	23/36 (64%)	12/28 (43%)	0.13 ^#^

* ANOVA test; ^#^ Chi-squared test.

## Data Availability

Data presented in this study are available from the corresponding author J.P.R. upon request. The data are not publicly available due to restrictions in accordance with consent provided by participants on the use of confidential data.

## Supplementary Materials

The following are available online at https://www.mdpi.com/article/10.3390/cancers13092079/s1, Table S1: Values of the hematological parameters in HNSCC patients prior to induction chemotherapy (ICT) treatment. Figure S1. Disease-specific (A) and overall (B) survival curves categorized according to the response to induction chemotherapy (ICT). Figure S2: ROC curves used to calculate the optimal cut-off values for the neutrophil-to-lymphocyte ratio (NLR) in relation to the response to induction chemotherapy (A) and patient survival (B). The optimal values calculated are shown. Figure S3: Disease-specific survival curves categorized according to neutrophil-to-lymphocyte ratio (NLR; A, B), PD-L1 composite proportion score (CPS; C,D), and CD8+ TIL density (E,F), in the subsets of patients treated with chemoradiotherapy or surgery. Figure S4: Disease-specific (A) and overall survival curves categorized according to the type of immune tumor microenvironment.
