# Supplementary material for: Immune Cell Infiltrates and Neutrophil-to-Lymphocyte Ratio in Relation to Response to Chemotherapy and Prognosis in Laryngeal and Hypopharyngeal Squamous Cell Carcinomas
Source: Cancers (Basel). 2021 Apr 25;13(9):2079. doi: 10.3390/cancers13092079 (PMC8123315; doi:10.3390/cancers13092079)
Supplement: Supplementary file 1 [file cancers-13-02079-s001.zip › cancers-1170820-supplementary.pdf]

SUPPLEMENTARY INFORMATION

Supplementary Table S1. Values of the hematological parameters in HNSCC patients prior to induction chemotherapy (ICT) treatment.

	**Hemoglobin (gr/dl)**	**Leucocytes (cells/mm^3)**	**Neutrophils (cells/mm^3)**	**Lymphocytes (cells/mm^3)**	**Platelets (cells/mm^3)**	**NLR**
**Mean**	14.3	8845.57	5847.21	2079.67	247183.33	3.69
**Median**	14.5	8400	5450	1920	240500	2.64
**SD**	1.64	1875.87	1946.26	880.25	66343.86	2.98
**Minimum**	10.6	6000	2880	500	147000	0.58
**Maximum**	17.6	13250	10700	4970	489000	17.4

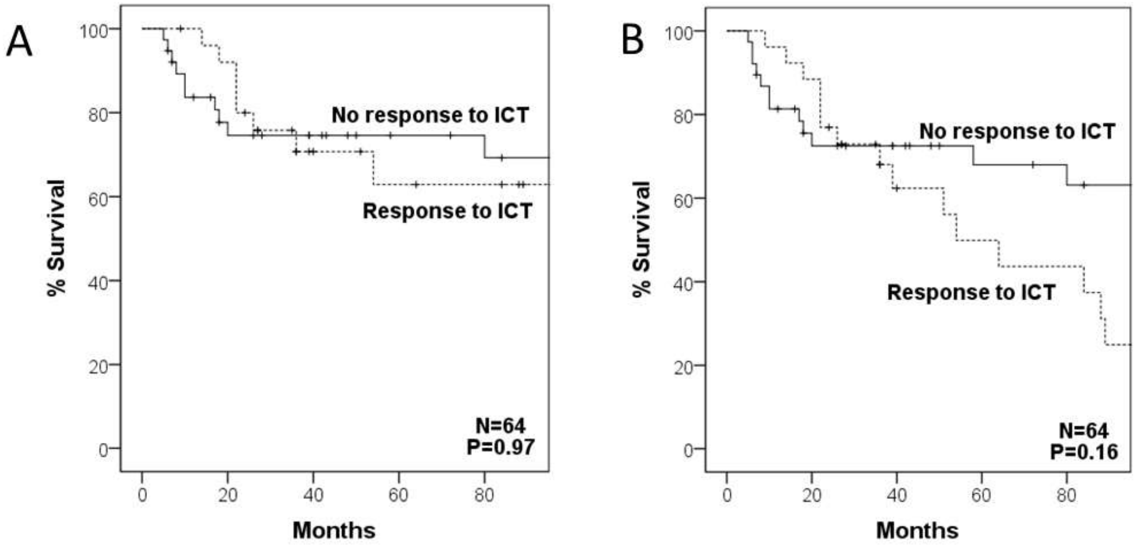

Supplementary Figure S1. Disease-specific (A) and overall (B) survival curves categorized according to the response to induction chemotherapy (ICT).

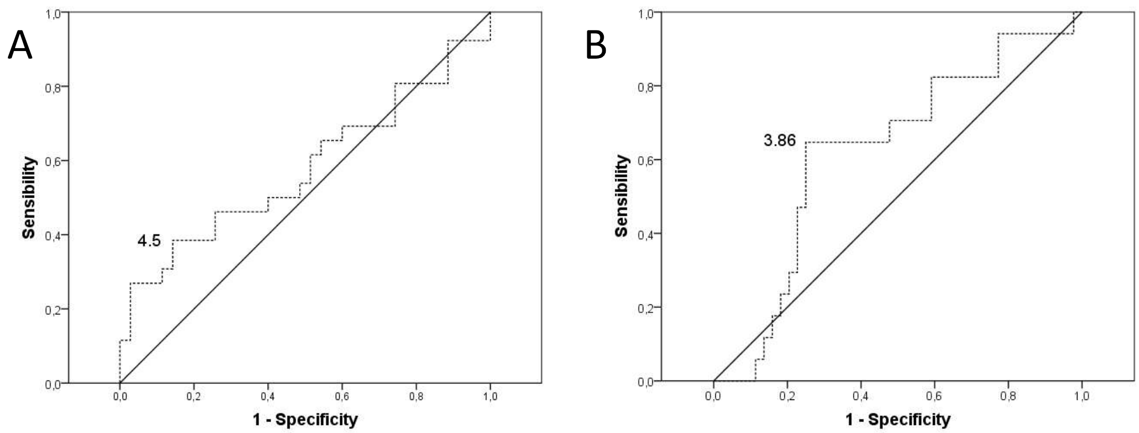

Supplementary Figure S2. ROC curves used to calculate the optimal cut-off values for the neutrophil-to-lymphocyte ratio (NLR) in relation to the response to induction chemotherapy (A), and patient survival (B). The optimal values calculated are shown.

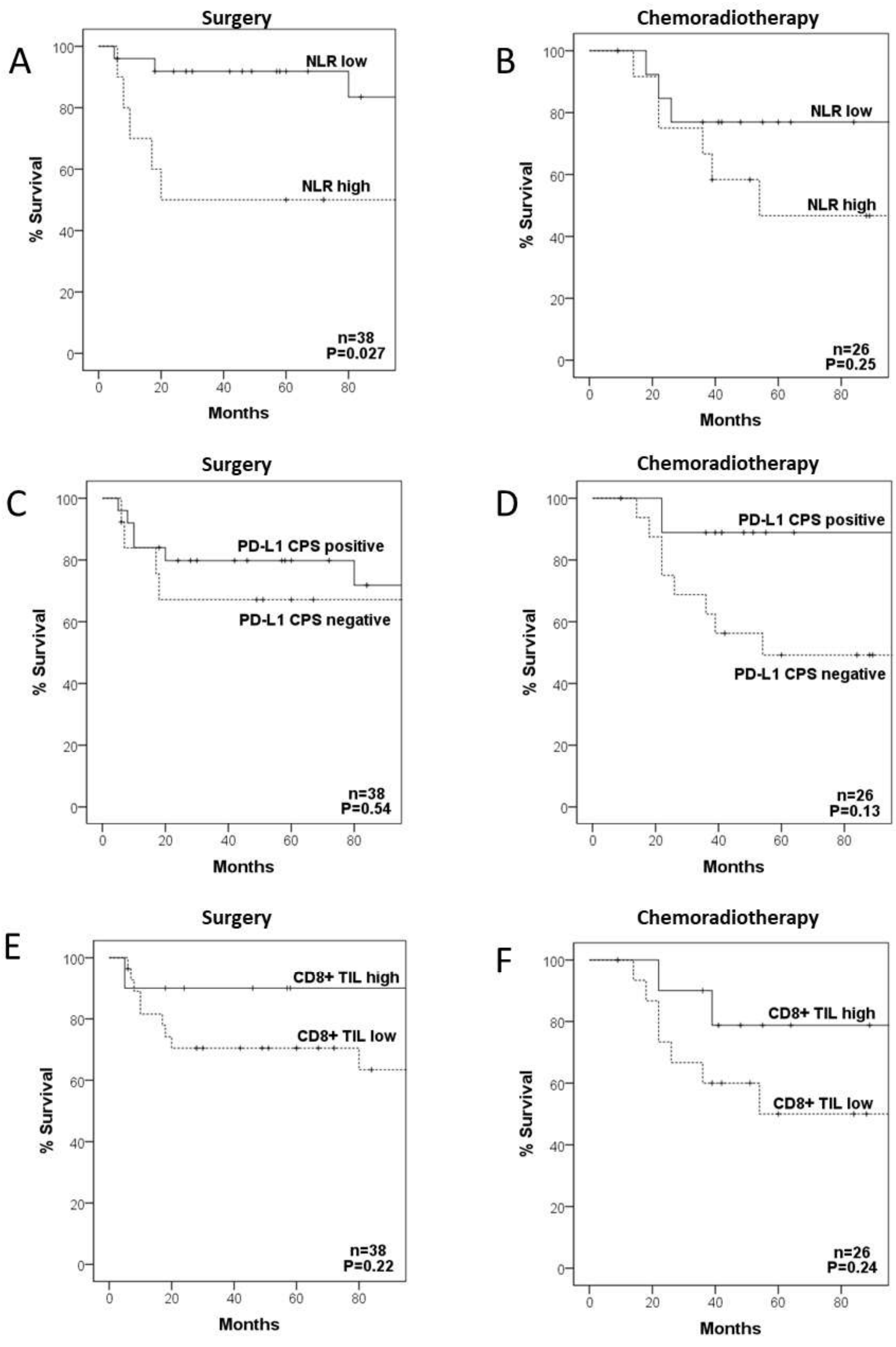

Supplementary Figure S3. Disease-specific survival curves categorized according to neutrophil-to-lymphocyte ratio (NLR; A, B), PD-L1 composite proportion score (CPS; C, D), and CD8+ TIL density (E, F), in the subsets of patients treated with chemoradiotherapy or surgery.

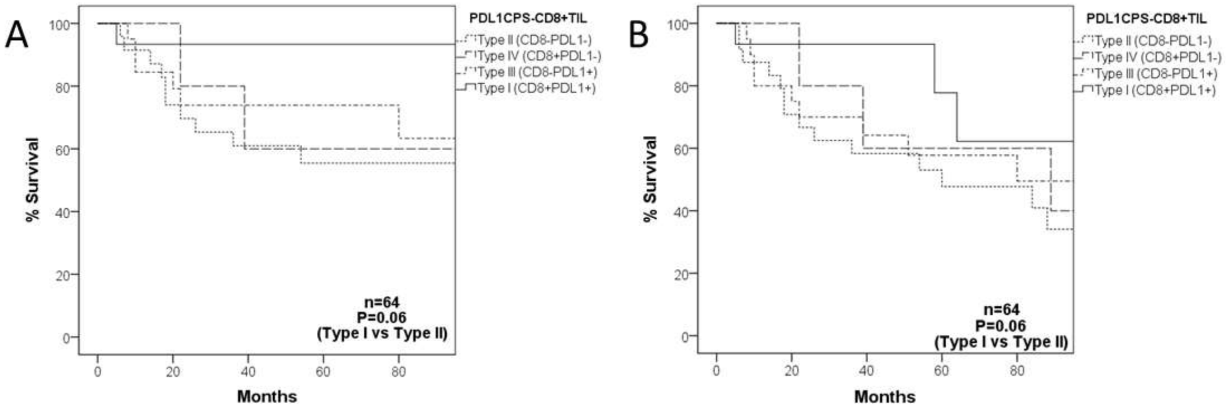

Supplementary Figure S4. Disease-specific (A) and overall survival curves categorized according to the type of immune tumor microenvironment.
